# Subtyping late-life depression according to inflammatory and metabolic dysregulation: a prospective study

**DOI:** 10.1017/S0033291720002159

**Published:** 2022-02

**Authors:** K. J. E. Kokkeler, R. M. Marijnissen, K. J. Wardenaar, D. Rhebergen, R. H. S. van den Brink, R. C. van der Mast, R. C. Oude Voshaar

**Affiliations:** 1 Department of Old Age Psychiatry, ProPersona, Arnhem, Wolfheze, The Netherlands; 2 University Center of Psychiatry & Interdisciplinary Center for Psychopathology of Emotion Regulation, University Medical Center Groningen, University of Groningen, Groningen, The Netherlands; 3 Department Psychiatry, GGZinGeest, Amsterdam Public Health Research Institute, VU University Medical Center, Amsterdam, The Netherlands; 4 Department of Psychiatry, Leiden University Medical Center, Leiden, The Netherlands; 5 Department of Psychiatry, CAPRI-University of Antwerp, Antwerp, Belgium

**Keywords:** Aged, depression, inflammation, latent class analysis, metabolic syndrome

## Abstract

**Background:**

Inflammation and metabolic dysregulation are age-related physiological changes and are associated with depressive disorder. We tried to identify subgroups of depressed older patients based on their metabolic-inflammatory profile and examined the course of depression for these subgroups.

**Methods:**

This clinical cohort study was conducted in a sample of 364 depressed older (⩾60 years) patients according to DSM-IV criteria. Severity of depressive symptoms was monitored every 6 months and a formal diagnostic interview repeated at 2-year follow-up. Latent class analyses based on baseline metabolic and inflammatory biomarkers were performed. Adjusted for confounders, we compared remission of depression at 2-year follow-up between the metabolic-inflammatory subgroups with logistic regression and the course of depression severity over 2-years by linear mixed models.

**Results:**

We identified a ‘healthy’ subgroup (*n* = 181, 49.7%) and five subgroups characterized by different profiles of metabolic-inflammatory dysregulation. Compared to the healthy subgroup, patients in the subgroup with mild ‘metabolic and inflammatory dysregulation’ (*n* = 137, 37.6%) had higher depressive symptom scores, a lower rate of improvement in the first year, and were less likely to be remitted after 2-years [OR 0.49 (95% CI 0.26–0.91)]. The four smaller subgroups characterized by a more specific immune-inflammatory dysregulation profile did not differ from the two main subgroups regarding the course of depression.

**Conclusions:**

Nearly half of the patients with late-life depressions suffer from metabolic-inflammatory dysregulation, which is also associated with more severe depression and a worse prognosis. Future studies should examine whether these depressed older patients benefit from a metabolic-inflammatory targeted treatment.

## Introduction

Depression is a common and disabling disorder in later life (Kok & Reynolds, 2017), and has a poorer prognosis compared with depression in younger individuals (Jeuring et al., 2018; Schaakxs et al., 2018). Among other factors, this worse prognosis of late-life depression (LLD) might be explained by aging-related physiological changes such as the occurrence of metabolic syndrome (MetS) and inflammation (Dowlati et al., 2010; Howren, Lamkin, & Suls, 2009; Koponen, Jokelainen, Keinänen-Kiukaanniemi, Kumpusalo, & Vanhala, 2008; Köhler et al., 2017; Marijnissen et al., 2013; Repousi, Masana, Sanchez-Niubo, Haro, & Tyrovolas, 2018).

MetS and depression have a bidirectional association. MetS predicts the onset and persistence of depression (Marijnissen et al., 2017; Vogelzangs et al., 2014a, Vogelzangs, Comijs, Oude Voshaar, Stek, & Penninx, 2014b), and vice versa depression the onset of MetS (Pan et al., 2012). The association between MetS and depression may be explained by immune-inflammatory alterations, which are present in both conditions (Capuron et al., 2008; Martinac et al., 2017). Meta-analyses consistently demonstrate an association between depression and inflammation, also in the absence of systemic inflammatory diseases (Capuron et al., 2008; Dowlati et al., 2010; Howren et al., 2009; Köhler et al., 2017). A proposed explanation for the association between inflammation and depression is the ‘cytokine hypothesis’, in which depression is considered to be the result of stress-related increased production of pro-inflammatory cytokines (Schiepers, Wichers, & Maes, 2005; Smith, 1991), which eventually leads to less availability of serotonin and over-activation of the hypothalamic, pituitary, and adrenal axis (Maes, Leonard, Myint, Kubera, & Verkerk, 2011; Wichers & Maes, 2004). This association, however, is less consistent in older populations than in younger populations (Vogelzangs et al., 2014a, 2014b). Moreover, metabolic dysregulations without inflammation have also been found in depressed older patients (Capuron et al., 2008). Nonetheless, depressed older persons with inflammation tend to have a worse prognosis (Gallagher, Kiss, Lanctot, & Herrmann, 2017) and higher depression symptom levels over time (Au, Smith, Gariépy, & Schmitz, 2015) than those without inflammation.

The inconsistent results in older samples might be explained by the etiological heterogeneity among LLD patients. Data-driven cluster analyses applied to MetS and inflammatory markers may identify etiologically distinct subgroups of patients with LLD. Until now, subtyping depression using data-driven clustering techniques has predominantly been based on patients' depressive symptom profiles (Marquand, Wolfers, Mennes, Buitelaar, & Beckmann, 2016; van Loo, de Jonge, Romeijn, Kessler, & Schoevers, 2012). In population-based studies, latent class analyses (LCA) have mostly yielded different classes (or subtypes) reflecting different levels of depressive symptom severity (e.g. Mezuk & Kendler, 2012; Ten Have *et al*. 2016), whereas only a few studies yielded classes with different symptom patterns (e.g. Wanders *et al*. 2016; Wardenaar, Wanders, Ten Have, de Graaf, & de Jonge, 2017). In clinical samples of depressed patients, LCAs have mostly yielded classes with different symptom profiles, such as melancholic severe depression and atypical severe depression, in both younger (Lamers et al., 2010; Li et al., 2014) and older patients (Veltman et al., 2017). Nonetheless, the atypical subtype was only associated with higher levels of inflammatory parameters among younger (Lamers et al., 2013), but not among older patients (Veltman et al., 2018). An explanation might be that (atypical) depression early in life is closely associated with metabolic-inflammatory dysregulation, whereas in later life atypical depression has a more heterogeneous etiology. To our knowledge, the only study that applied LCA on somatic biomarkers was conducted in depressed younger (18–65 years) patients and showed that the overweight subgroup had the highest levels of psychopathology (Beijers et al., 2018).

The first objective of the present study was to classify depressed older patients according to their patterns of metabolic and inflammatory dysregulations. We hypothesized to identify a subgroup free of metabolic-inflammatory dysregulation and one or more subgroups with a specific metabolic-inflammatory profile. Our second objective was to compare the prognosis of depression over a 2-year follow-up between identified subgroups. We hypothesized to find a worse prognosis for subgroups characterized by metabolic-inflammatory dysregulation.

## Methods

### Study design and sample

The study is embedded in the Netherlands Study of Depression in Older persons (NESDO), an ongoing multi-site cohort study designed to examine the course and consequences of depressive disorders in older persons (⩾ 60 years) (Comijs et al., 2011, 2015). In brief, the cohort consists of 378 depressed and 132 non-depressed older persons aged from 60 to 93 years, recruited from mental health care institutes and general practitioners from 2007 until 2010. Participants with a primary diagnosis of dementia, a Mini Mental State Examination (MMSE) score of <18 (out of 30 points) (Folstein, Folstein, & McHugh, 1975), a primary psychotic or bipolar disorder or insufficient command of the Dutch language were excluded. Inclusion was based on the diagnosis of depression according to the Diagnostic and Statistical Manual of Mental Disorders-IV-R (DSM-IV-R) criteria and was defined as a past 6-months major depressive disorder (MDD) (95%), dysthymic disorder (26.5%) or past-month minor depression (two to four depressive symptoms lasting at least 2 weeks) (5%). The comparison group of non-depressed participants was recruited at the same general practices that recruited the depressed participants and had no history of depressive disorder.

Baseline assessments included written questionnaires, a face-to-face interview, a medical examination, cognitive tests and the collection of fasten blood samples in the morning. Information was gathered about mental health outcomes, demographic characteristics and psychosocial, biological, cognitive and genetic determinants. At 2-year follow-up, all measures open to change were administered again (Comijs et al., 2015). Furthermore, the course of depression was assessed every 6-months using postal questionnaires.

The ethical review boards of the participating institutes approved this study and all participants provided written informed consent.

For the present study, we selected only the depressed patient group (*n* = 378). A total of 14 patients were excluded because of a fever in the week prior to blood withdrawal, as this may bias the level of inflammatory markers. Of the remaining 364 patients, 273 patients participated in the 2-year follow-up visit.

### Assessment of depression

At baseline and 2-year follow-up, the Composite International Diagnostic Interview (CIDI; WHO version 2.1) was used to assess whether criteria were met for a diagnosis of depression or dysthymia, according to the DSM-IV-R criteria. The CIDI is a structured clinical interview with high validity for depressive and anxiety disorders (Kessler et al., 2010; Wittchen et al., 1991). Questions were added to diagnose current minor depression according to the research criteria of the DSM-IV-R (Comijs et al., 2011).

Severity of depression was measured with the well-validated 30-item Inventory of Depressive Symptomatology-Self Report (IDS-SR; Hegeman *et al*., 2012; Rush, Gullion, Basco, Jarrett, & Trivedi, 1996). The course of depression severity was assessed using the repeated IDS-SR scores taken at baseline and every 6-months until 2-year follow-up (five IDS-SR scores per participant).

Patients who, after 2-year follow-up, no longer met the DSM-IV-TR criteria for depression (either 6-months MDD or dysthymic disorder, or past-month minor depression) were considered remitted from depression.

### Biomarkers

Several metabolic and inflammatory biomarkers were used as input variables for the LCA to identify biomarker-based subgroups of patients.

#### Metabolic dysregulation

Seven included markers of metabolic dysregulation were waist circumference, levels of triglycerides, high-density lipoprotein (HDL), low-density lipoprotein (LDL), glucose level, as well as systolic and diastolic blood pressure. Waist circumference was assessed in centimeters halfway between lower rib margin and the iliac crest following normal expiration upon light clothing. Triglyceride, HDL cholesterol, LDL cholesterol, total cholesterol and glucose levels were measured in morning fasting blood samples using routine standardized laboratorial methods. Blood pressure was averaged over two readings measured in a supine position using an electric Omron sphygmomanometer. All variables were considered as continuous measures in the LCA.

Body mass index (BMI) and total cholesterol were not included as these markers were strongly (*r* > 0.60) correlated with other markers (*r* = 0.79 for BMI and waist circumference; *r* = 0.92 for total- and LDL-cholesterol). The correlations between included variables varied between −0.42 and 0.59.

#### Inflammation

Four inflammation markers, i.e. high-sensitive C-reactive protein (hsCRP), interleukin-6 (IL-6), Growth Differentiation Factor-15 (GDF-15) and neutrophil gelatinase-associated lipocalin (NGAL), were assessed in fasting blood samples obtained in the morning between 8 and 9 a.m. after an overnight fasting, and kept frozen at −80 °C. These markers have previously been observed to be associated with depression (Dowlati et al., 2010; Howren et al., 2009; Köhler et al., 2017; Naudé et al., 2013; Teunissen, Durieux-Lu, Blankenstein, Oude Voshaar, & Comijs, 2016). All markers were assessed using well-validated assays. hsCRP levels were measured in duplicate by using an immunoturbidimetric assay, IL-6 and NGAL by using an enzyme-linked immunosorbent assay and GDF-15 using an automated assay on Abbott Architect. Intra- and inter-assay coefficients of variation were 2% and 2% for hsCRP, 8% and 12% for IL-6 and 3% and 5% for NGAL. The intra-assay coefficient of variation for GDF-15 was 3.8%.

### Patient characteristics

Several additional measurements were performed to compare any identified LCA classes.

#### Metabolic dysregulation

In addition to the MetS variables used in the LCA, we assessed the presence of MetS (yes/no), diabetes mellitus (yes/no), BMI and total cholesterol at baseline. The MetS was defined according to the National Cholesterol Education Programme (NCEP)-Adult Treatment Panel III guidelines (Grundy et al., 2004).

#### Clinical characteristics

Key clinical characteristics of interest were: age of onset of depressive disorder (CIDI question), depression severity based on IDS-SR sum score, melancholic and/or atypical features [presence of DSM-defined melancholic features were assessed based on the IDS-items according to Kahn's algorithm (Khan et al., 2006) and DSM-defined atypical features based on IDS-items according to Novick's algorithm (Novick et al., 2005)]. Furthermore, mood, motivation and somatic-affective symptom dimensions according to the IDS subscale scores (Hegeman et al., 2012), diagnosis of dysthymia according to DSM-IV-R criteria, a current anxiety disorder (CIDI), level of anxiety based on the Beck Anxiety Inventory (BAI) sum scores and the use of antidepressants (yes/no, as well as differentiated into categories SSRI/TCA/MAO/other).

#### Demographics

Standard demographics included age, sex, educational level (in years) and marital status (partner yes/no).

#### Life-style and physical health indicators

Indicators of life style and physical health included smoking (yes/no), use of alcohol, physical activity, global cognitive functioning and number of chronic diseases.

The number of alcoholic drinks per day was based on the first two items of the Alcohol Use Disorders Identification Test (AUDIT) (Aalto, Alho, Halme, & Seppä, 2011; Babor, Kranzler, & Lauerman, 1989). Physical activity was measured with the last-seven-days short-form (8-items) of the International Physical Activity Questionnaire (IPAQ) and classified as minimal, moderate or high (Craig et al., 2003). Global cognitive functioning was assessed by the MMSE (Folstein et al., 1975). The number of chronic diseases was assessed by self-report questions with high accuracy compared to general practitioner information (Kriegsman, Penninx, van Eijk, Boeke, & Deeg, 1996). We asked for cardiac disease (including myocardial infarction), peripheral atherosclerosis, stroke, diabetes mellitus, chronic obstructive pulmonary disease (asthma, chronic bronchitis or pulmonary emphysema), arthritis (rheumatoid arthritis or osteoarthritis), cancer or any other disease.

### Statistical analysis

First, LCAs were performed to identify biomarker-based patient subgroups. Models with increasing numbers of classes were fit to the data and the most optimal model was selected based on the lowest values for the Bayesian information criterion (BIC) and Akaike information criterion (AIC). In addition, model interpretability (i.e. sufficient qualitative differentiation between classes) was considered when choosing the number of classes. The LCAs were run using a robust maximum likelihood estimator (MLR) in Mplus 5.0 (Muthén & Muthén, 2007). To avoid a model solution at a local maximum, each model was run with multiple random starts. After identification of the optimal model, each patient was allocated to a class based on their highest posterior class probability.

After class-assignment, the subgroups were compared with respect to demographic and clinical characteristics at baseline. Next, we examined the association of class-membership at baseline (independent variable) with remission of LLD at 2-year follow-up (dependent variable) using logistic regression. Finally, the course of depressive symptoms over time as assessed with the 6-monthly IDS-SR was examined in the different classes using mixed model analysis (Twisk, de Boer, de Vente, & Heymans, 2013). Models with random coefficients for intercept and/or slope per subject were compared, and the following elements were subsequently tested to determine the best fitting model, using the likelihood ratio test: (1) a general linear change in depression severity in all classes, (2) a general quadratic – i.e. parabolic – change over time, (3) stable differences in depression severity between the classes and (4) differences in depression course between the classes. All models were adjusted for covariates known to be associated with the course of depression, i.e. age, sex, level of education, cognitive functioning (MMSE), number of chronic diseases, antidepressant drug use and lifestyle characteristics (i.e. use of alcohol, smoking and physical activity).

These analyses were conducted in SPSS, version 25. We considered *p* values less than 0.05 as significant.

## Results

### Characteristics of the study population

The 364 depressed older patients had a mean age of 70.7 (standard deviation 7.3; range 60–90) years and 66.2% were females (Tables 1 and 2).

**Table 1.** Metabolic and inflammatory differences between subgroups at baseline

	Total	Class A	Class B	Class C	Class D			Post hoc
	sample^a^ (*N* = 364)	Healthy (*n* = 181)	Metabolic and inflammatory (*n* = 137)	Severe inflammation (*n* = 27)	Mild inflammation (*n* = 14)	*p*-value^b^	*F*/χ^2^	Hochberg^c^/χ^2^
Metabolic dysregulation								
Waist circumference, mean (s.d.)	93.6 (13.1)	85.5 (9.0)	103.0 (11.3)	97.3 (11.8)	98.5 (11.5)	**<0.001**	77.6	A < B, C, D
Triglyceride, mean (s.d.)^d^	1.32 (1.58)	1.05 (1.39)	1.82 (1.53)	1.28 (1.55)	1.34 (1.50)	**<0.001**	52.4	B > A, C, D
HDL, mean (s.d.)^d^	1.50 (1.33)	1.74 (1.28)	1.27 (1.25)	1.38 (1.31)	1.36 (1.16)	**<0.001**	49.0	A > B, C, D
LDL, mean (s.d.)	3.36 (1.05)	3.48 (1.04)	3.36 (1.06)	3.01 (0.99)	2.61 (1.00)	**0.006**	4.2	D < A
Cholesterol, mean (s.d.)^e^	5.47 (1.19)	5.70 (1.12)	5.39 (1.22)	4.98 (1.22)	4.59 (1.23)	**<0.001**	6.6	A > C, D
Systolic blood pressure, mean (s.d.)	147 (20)	140 (18)	156 (20)	148 (18)	140 (17)	**<0.001**	20.1	B > A, D
Diastolic blood pressure, mean (s.d.)	81 (11)	77 (9)	87 (10)	80 (11)	74 (14)	**<0.001**	28.9	B > A, C, D
Glucose, mean (s.d.)^d^	5.74 (1.21)	5.42 (1.14)	6.17 (1.25)	5.86 (1.19)	5.68 (1.22)	**<0.001**	13.3	B > A
BMI, mean (s.d.)^e^	26.3 (4.4)	24.3 (3.3)	28.8 (4.5)	27.6 (4.3)	25.5 (5.1)	**<0.001**	35.3	B > A, D, C > A
Diabetic (%)^e^	12.9	5.0	21.1	14.8	28.6	**<0.001**	21.6	A < B, C, D
MetS (%)^e^	43.7	18.8	75.2	55.6	42.9	**<0.001**	102.2	A < B, C, D, B > A, C, D
Inflammation								
hsCRP, mean (s.d.)^d^	1.90 (3.21)	1.16 (2.60)	2.02 (2.32)	16.66 (1.27)	2.92 (2.94)	**<0.001**	73.8	C > A, B, D, A < B, C, D
IL-6, mean (s.d.)^d^	0.81 (2.91)	0.57 (2.23)	0.85 (2.78)	2.65 (2.41)	1.27 (3.18)	**<0.001**	24.9	A < B, C, D, C > A, B
GDF-15, mean (s.d.)^d^	906.78 (1.61)	733.50 (1.39)	1006.47 (1.50)	1045.92 (1.51)	3246.38 (1.36)	**<0.001**	80.2	D > A, B, C, A < B, C, D
NGAL, mean (s.d.)^d^	58.34 (1.48)	50.76 (1.41)	64.45 (1.42)	59.14 (1.31)	122.43 (1.58)	**<0.001**	34.2	D > A, B, C, A < B, D

a Total sample consisting of six classes.

b *p* value from ANOVA or χ^2^.

c Hochberg post hoc analysis was chosen because of differences in class-size.

d Log-transformation was performed, the values listed are the retransformed mean and s.d. of the log values.

e Parameters not included in the LCA.

**Table 2.** Demographic and clinical differences between subgroups at baseline

	Total sample^a^ (*N* = 364)	Class A (*n* = 181)	Class B (*n* = 137)	Class C (*n* = 27)	Class D (*n* = 14)	*p* value ANOVA/χ^2^	Post hoc Hochberg/χ^2^
Demographics							
Sex, female (%)	66.2	79.0	53.3	66.7	35.7	**<0.001**	A > B, D
Age mean (s.d.), years	70.7 (7.3)	70.3 (6.9)	70.4 (7.7)	71.4 (6.3)	78.4 (8.3)	**0.001**	D > A, B, C
Years of education, mean (s.d.), years	10.4 (3.4)	10.8 (3.3)	10.0 (3.5)	10.5 (3.5)	9.2 (3.8)	0.088	
Partner or married (%)	53.0	51.4	57.7	48.1	42.9	0.529	
Mental/physical health							
Current smoker (%)	25.5	23.0	27.7	25.9	35.7	0.637	
No. of alcohol consumptions/day, mean (s.d.)	0.56 (0.91)	0.54 (0.89)	0.58 (0.93)	0.58 (0.80)	0.38 (0.76)	0.862	
Physical activity							
Low (%)	30.9	22.9	37.9	29.6	71.4	**0.002**	D ≠ A, B, C
Moderate (%)	38.0	40.4	36.4	40.7	21.4		A ≠ B
High (%)	31.2	37.1	25.8	29.6	7.1		
MMSE, mean (s.d.)	27.7 (2.0)	27.8 (1.9)	27.6 (2.2)	28.0 (1.8)	26.8 (1.9)	0.197	
No. of chronic disease, mean (s.d.)	2.5 (1.7)	2.2 (1.7)	2.9 (1.6)	2.7 (1.5)	3.2 (1.9)	**0.001**	B > A
Clinical characteristics							
MDD past 6 months (%)	94.8	94.5	95.6	92.6	92.9	0.900	
Age of onset mean (s.d.), years	48.8 (20.4)	50.6 (19.7)	45.6 (20.0)	46.2 (21.7)	63.9 (22.2)	**0.005**	D > B, C
Dysthymia past 6 months (%)	26.1	21.0	36.5	11.1	21.4	**0.004**	B > A, C
Severity (IDS), mean sum score (s.d.)	29.8 (12.9)	27.7 (12.6)	32.7 (13.3)	29.6 (11.5)	30.4 (14.2)	**0.009**	B > A
Depression type (%)							
No atypical/melancholic	79.7	84.7	72.2	81.5	78.6	0.133	
Atypical	7.3	5.1	9.0	7.4	21.4		
Melancholic	11.0	8.5	15.8	11.1	–		
Atypical and melancholic	2.0	1.7	3.0	–	–		
Depression subscale (IDS)							
Mood, mean (s.d.)	8.8 (5.1)	7.9 (5.1)	10.2 (5.0)	8.6 (4.6)	8.9 (5.4)	**0.002**	B > A
Motivation, mean (s.d.)	5.0 (3.1)	4.5 (3.0)	5.5 (3.3)	5.1 (3.0)	5.6 (3.8)	**0.033**	B > A
Somatic, mean (s.d.)	9.7 (4.2)	9.3 (4.0)	10.2 (4.3)	9.8 (4.4)	9.9 (4.4)	0.233	
Anxiety disorder past 6 months (CIDI) (%)	37.9	40.3	38.0	33.3	14.3	0.260	
Level of anxiety (BAI), mean sum score (s.d.)	17.2 (11.1)	15.6 (10.6)	19.5 (11.8)	15.6 (9.3)	18.8 (11.1)	**0.020**	B > A
Antidepressant use							
Any (%)	72.3	70.7	75.2	70.4	57.1	0.495	
SSRI (%)	27.5	32.0	24.3	25.9	7.1	0.138	
TCA (%)	22.0	22.2	25.5	11.1	7.1	0.198	
MAO (%)	0.5	0.6	0.7	0.0	0.0	0.959	
Other (%)	28.1	21.5	31.6	37.0	50.0	**0.028**	A < B, D

Class A = healthy subgroup, class B = metabolic and inflammatory dysregulation subgroup, class C = severe inflammation, class D = mild inflammation.

a Total sample consisting of six classes.

### Latent class analyses (LCA)

The best fitting LCA model according to the AIC and BIC was the six-class model, which also had good interpretability. Two classes were not used in the subsequent analyses, since these classes were too small and based on an extreme value of only one specific parameter, i.e. hsCRP (class 5, three patients) and IL-6 (class 6, two patients).

Furthermore, a sensitivity analysis, performed by repeating the LCA restricted to the subsample of patients with a current (6-month) MDD (*n* = 345), resulted in a comparable class solution.

Table 1 shows the metabolic and inflammation parameters of the four selected classes (A through D). Class A (*n* = 181, 49.7%) consisted of a relatively healthy subgroup of depressed patients with low scores across MetS and inflammation markers and is referred to as ‘Healthy’ (healthy solely referring to their metabolic-inflammatory status). This depressed subgroup was characterized by comparatively lower measures of waist circumference, triglyceride, glucose and BMI, a lower frequency of diabetes and the MetS, and higher levels of HDL cholesterol whereas inflammation markers were not elevated.

In class B (*n* = 137, 37.6%), 75.2% of the patients had MetS, reflected by higher waist circumference, triglyceride levels and systolic and diastolic blood pressure; as well as slightly higher levels of inflammatory markers. Therefore, this subgroup is referred to as ‘Metabolic and inflammatory dysregulation’.

Class C (*n* = 27, 7.4%) was characterized by higher levels of inflammation markers, mostly hsCRP and IL-6. This subgroup is referred to as ‘Severe inflammation’.

Similarly, class D (*n* = 14, 3.8%) showed increased inflammation levels, although less severe than the previous group, and was characterized by elevated measures of GDF-15 and NGAL. This subgroup is referred to as ‘Mild inflammation’.

### Characteristics of LCA identified subgroups

Table 2 shows the demographical and clinical characteristics of the four depressed subgroups (A through D, *n* = 359). The *healthy* subgroup (class A) predominantly consisted of physically active females with few chronic diseases and less severe levels of depression and anxiety than the other classes. In contrast, the *metabolic and inflammatory dysregulation* subgroup (class B) was less physically active, suffered from more chronic diseases and had more severe levels of depression and anxiety than the other subgroups. The higher depression severity in this class was based on higher scores on the mood and motivation items compared to the other classes. There was no difference in frequency of atypical or melancholic depression subtype between the classes. However, this *metabolic and inflammatory dysregulation* subgroup more frequently suffered from dysthymia. The *severe inflammation* subgroup (class C) did not distinguish itself from the other subgroups on demographic or clinical parameters. The *mild inflammation* subgroup (class D) was the oldest and less physically active subgroup with a higher age of depression onset than the *metabolic and inflammatory dysregulation* subgroup (class B) and the *severe inflammation* (class C) subgroup, and was the only predominantly male group.

### Remission of late-life depression at 2-year follow-up

Follow-up data were missing for 86/359 patients (24.0%). In total 23/86 patients were deceased at 2-year follow-up, the rest was lost in follow-up because of different reasons. The dropout-rate differed significantly between the depressed subgroups (χ^2^ = 10.0, df = 3, *p* = 0.018). The *mild inflammation* subgroup (class D) had the highest dropout-rate [7/14 (50.0%), of which three deceased), followed by the *severe inflammation* subgroup [class C, 8/27 (29.6%), of which three deceased], the *metabolic and inflammatory dysregulation* subgroup [class B, 38/137 (27.7%), of which 11 deceased] and the *healthy* subgroup [class A, 33/181 (18.2%), of which six deceased].

Patients who no longer participated at 2-year follow-up differed from the other patients in several baseline metabolic, inflammation, demographic and clinical parameters. Drop-outs had a higher waist circumference [96.4 (13.7) *v.* 92.6 (12.9); *t* = −2.4, df = 356, *p* = 0.019), a higher BMI [27.2 (4.7) *v.* 26.0 (4.3); *t* = −2.2, df = 357, *p* = 0.026], a higher level of hsCRP [2.31 (2.87) *v.* 1.71 (3.12); *t* = −2.1, df = 346, *p* = 0.037], a higher level of GDF-15 [1080.19 (1.71) *v.* 856.25 (1.56); *t* = −3.9, df = 349, *p* ⩽ 0.001] and were less physically active (low 45.2%, moderate 27.4%, high 27.4% *v.* low 26.5%, moderate 41.3%, high 32.2%; χ^2^ = 10.9, df = 2, *p* = 0.004) than patients whom did not drop-out.

At 2-year follow-up 141/273 (51.6%) patients did no longer meet the DSM-IV-TR criteria for depression (either 6-month MDD or dysthymic disorder, or past-month minor depression). There was a significant difference in remission-rates between the four depressed subgroups (χ^2^ = 12.2, df = 3, *p* = 0.007). The *healthy* subgroup (class A) had the highest remission rate (90/148, 60.8%), followed by the *mild inflammation* subgroup (class D, 4/7, 57.1%) and the *severe inflammation* subgroup (class C, 9/19, 47.4%). The *metabolic and inflammatory dysregulation* subgroup had the lowest remission rate (class B, 38/99, 38.4%).

Logistic regression confirmed that class membership predicted 2-year remission (Wald = 11.9, df = 3, *p* = 0.008), but the association was no longer significant after adjustment for covariates (Wald = 6.3, df = 3, *p* = 0.098), although the difference between the two largest subgroups remained. Patients in the *metabolic and inflammatory dysregulation* subgroup (class B) had lower odds of achieving remission compared to patients in the *healthy* subgroup (class A), in both the unadjusted [odds ratio (OR) 0.40 (95% confidence interval (CI) 0.24–0.67), *p* = 0.001] and adjusted analysis [OR 0.49 (95% CI 0.26–0.91), *p* = 0.025].

### Course of late-life depression over 2 years

As shown in Table 3, the successive inclusion of a linear effect of time, a quadratic effect of time, and a main effect of class improved the model fit of the mixed model exploring the severity of depressive symptoms over time as indicated by the likelihood ratio tests at each step. A further inclusion of either an interaction effect of class with time (step 4A) or with time square (step 4B), did not improve the model fit any further. This means that no significant differences were found between the classes in depression course. At all steps, a random intercept and random slope model proved to be the best fitting model, as indicated by the likelihood ratio test.

**Table 3.** Model selection and optimal mixed model analysis of course of depression severity over 2-year follow-up

Selection of optimal mixed model						
	Unadjusted			Adjusted^a^		
Consecutive steps & tested effect	χ^2^	df	*P*	χ^2^	df	*p*
1 – Linear effect of time	74.8	1	<0.001	66.6	1	<0.001
2 – Quadratic effect of time	14.3	1	<0.001	14.0	1	<0.001
3 – Main effect of class	25.6	3	<0.001	15.9	3	0.001
4A – Interaction of class with linear time	4.0	3	0.264	3.9	3	0.275
4B – Interaction of class with quadratic time	5.5	3	0.137	5.6	3	0.133
Best fitting model for course of depressive symptoms						
	Unadjusted			Adjusted^a^		
Effects	*B* (s.e.)	*F*/*t*	*P*	*B* (s.e.)	*F*/*t*	*p*
Class						
Class A ‘Healthy’ (*n* = 181)	Reference^b^					
Class B ‘Inflammatory-metabolic dysregulation’ (*n* = 137)	4.8 (1.2)	3.9	<0.001	3.1 (1.3)	2.3	0.021
Class C ‘Severe inflammation’ (*n* = 27)	4.1 (2.3)	1.8	0.070	3.1 (2.2)	1.4	0.162
Class D ‘Mild inflammation’ (*n* = 14)	3.2 (3.1)	1.0	0.300	1.0 (3.2)	0.3	0.747
Time (years since baseline)	−6.2 (0.9)	42.8	<0.001	−6.0 (0.9)	40.4	<0.001
Time^2^	1.9 (0.5)	17.9	<0.001	1.8 (0.5)	16.7	<0.001

a Adjusted for age, sex, years of education, smoking, alcohol use, physical activity, somatic comorbidity, global cognitive functioning and use of antidepressants.

b No other significant differences between classes (*p* < 0.05) were found.

The optimal model showed that for all patient classes, depression severity is best estimated to decline linearly from baseline by (after adjustment for covariates) 6.0 points per year on the IDS-SR (95% CI 4.1–7.8), but this decline tapers off at a quadratic rate of 1.8 (1.0–2.7) points per squared number of years since baseline, resulting in an expected net decline of 4.3 points over the first year and 0.6 over the second year. Furthermore, the *metabolic and inflammatory dysregulation* subgroup (class B) consistently scored 4.8 (2.4–7.2) points above the *healthy* subgroup (class A), from baseline on, and this difference is only partly explained by the factors known to influence the course of depression which were included as covariates. After adjustment for these covariates, the stable difference between the two subgroups remained, but was reduced to 3.1 (0.5–5.6).

Because almost all change in depression severity occurred in the first year of follow-up, we also analyzed the course of depression over this period separately. Here too the models including both a random intercept and slope proved to be the best fitting models, at all steps. All consecutive additions of (1) a linear effect of time, (2) a quadratic effect of time, (3) a main effect of class and (4) an interaction effect of class with either time (4A) or time square (4B) improved the fit of the model (see Table 4). Because in step 4 the addition of the interaction between class and time improved the fit of the model more than the addition of interaction between class and time square (as indicated by the reduction in log likelihood), this model was chosen as best fitting model for the course of depression severity over the first follow-up year.

**Table 4.** Model selection and optimal mixed model analysis of course of depression severity over the first year of follow-up

Selection of optimal mixed model						
	Unadjusted			Adjusted^a^		
Consecutive steps and tested effect	χ^2^	df	*p*	χ^2^	df	*p*
1 – Linear effect of time	51.5	1	<0.001	51.2	1	<0.001
2 – Quadratic effect of time	18.2	1	<0.001	16.2	1	<0.001
3 – Main effect of class	24.9	3	<0.001	14.8	3	0.002
4A – Interaction of class with linear time	14.0	3	0.003	14.8	3	0.002
4B – Interaction of class with quadratic time	10.8	3	0.013	12.6	3	0.006
Best fitting model for course of depressive symptoms						
	Unadjusted			Adjusted^a^		
Effects	*B* (s.e.)	*F*/*t*	*p*	*B* (s.e.)	*F*/*t*	*p*
Time (years since baseline)	−7.3 (3.1)	22.0	<0.001	−6.9 (3.0)	20.3	<0.001
Time^2^	8.2 (1.9)	18.4	<0.001	7.8 (1.9)	16.2	<0.001
Class						
Class C ‘Severe inflammation’ (*n* = 27)	Reference^b^	–	–	Reference^c^	–	–
Class A ‘Healthy’ (*n* = 181)	−1.7 (2.5)	0.7	0.513	−0.7 (2.4)	0.3	0.770
Class B ‘Inflammatory-metabolic dysregulation’ (*n* = 137)	2.8 (2.6)	1.1	0.287	1.6 (2.4)	0.7	0.512
Class D ‘Mild inflammation’ (*n* = 14)	0.2 (4.1)	0.1	0.957	−0.8 (3.9)	0.2	0.847
Class × Time:						
Class C × Time	Reference^c^	–	–	Reference^c^	–	–
Class A × Time	−6.3 (2.6)	2.6	0.017	−6.5 (2.5)	2.5	0.010
Class B × Time	−5.6 (2.7)	2.7	0.041	−5.1 (2.6)	0.2.0	0.052
Class D × Time	−0.6 (4.7)	4.7	0.902	−0.8 (4.5)	0.2	0.862

a Adjusted for age, sex, years of education, smoking, alcohol use, physical activity, somatic comorbidity, global cognitive functioning and use of antidepressants.

b Difference between class A and class B in unadjusted analysis (*t* = 3.163; *p* = 0.002), not in adjusted analysis (*t* = 1.590; *p* = 0.113).

c No other significant differences between classes (*p* < 0.05) were found.

**Fig. 1.**
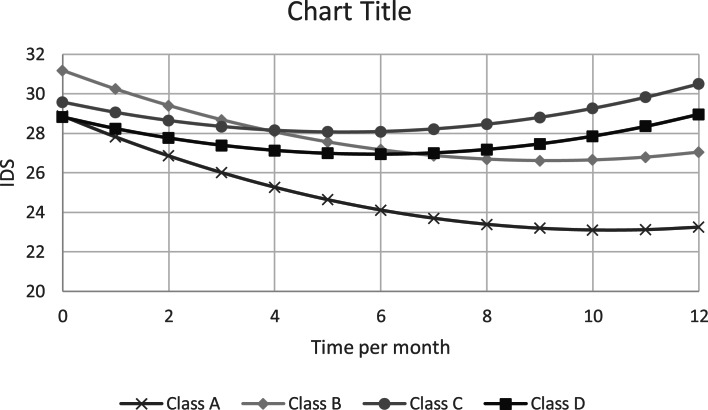
Estimated course of depression severity over the first year (adjusted for age, sex, years of education, smoking, alcohol use, physical activity, somatic comorbidity, global cognitive functioning and use of antidepressants). Class A: ‘Healthy’, class B: ‘Metabolic and inflammatory dysregulation’, class C: ‘Severe inflammation’, class D: ‘Mild inflammation’.

Table 4 and Fig. 1 show that for the *severe inflammation* subgroup (class C, reference group in Table 4) it can be seen, that – after adjustment for covariates – a sharp linear decline from baseline in depression severity occurred (at a rate of 6.9 points per year on the IDS-SR; 95% CI 0.9–12.8), but this decline soon leveled off at a quadratic rate of 7.8 (4.0–11.6) points per squared number of years since baseline, in such a way that after about five months a maximum decline of 1.5 points from the baseline level was reached, after which the severity rose again to above baseline level after 1 year. The course of depression in the *severe inflammation* subgroup (class C) did not differ significantly from that seen in the *mild inflammation* subgroup (class D). In the *metabolic and inflammatory dysregulation* (class B) and *healthy* (class A) subgroups, the decline in depression severity over the first follow-up year was significantly larger than in the *severe inflammation* subgroup (class C), with a difference of 5.1 (<−0.1 to 10.1) and 6.5 (1.6–11.5) points per year, respectively. Finally, a difference was seen in the unadjusted analysis between the *metabolic and inflammatory dysregulation* (class B) and *healthy* (class A) subgroups, with the latter scoring 4.4 (1.7–7.2) points lower on average at baseline, but this difference was no longer significant after correction for covariates.

## Discussion

### Main findings

Among 364 depressed older patients, LCA identified six classes according to metabolic-inflammatory parameters. The largest class consisted of 181 (49.7%) depressed older persons showing minimal metabolic-immune dysregulation. The other five groups did show deviations from this health pattern in their metabolic and/or inflammatory marker levels. The largest effected subgroup consisted of patients with an overall, but relatively mild level of metabolic-inflammatory dysregulation (*n* = 137, 37.6%). Smaller subgroups were characterized by increased levels of hsCRP and IL-6 (severe inflammation, *n* = 27, 7.4%), by increased levels of GDF-15 and NGAL (mild inflammation, *n* = 14, 3.8%), an isolated extremely elevated level of hsCRP (*n* = 3), or an isolated extremely elevated level of GDF-15 (*n* = 2). The three larger subgroups with low grade inflammation showed the highest levels of psychopathology compared to the healthy subgroup. This was most pronounced for the metabolic inflammatory dysregulation subgroup, which had the highest level of depression severity. In line with our hypothesis, patients in the subgroup with both metabolic and inflammatory dysregulation consistently scored higher on depression severity over the follow-up period than patients without metabolic-inflammatory dysregulation, and were less often remitted at 2 year follow-up. Patients with severe inflammation showed the least decline in depression severity in the first year.

### Metabolic and inflammatory characterized depression

To our knowledge, only one study applied LCA to identify depression subgroups within depressed younger (18–65 years) patients using somatic biomarkers, including metabolic and inflammatory markers. Three subgroups were identified that were labeled as ‘lean’, ‘average’ and ‘overweight’ based on their average BMI value. However, this labeling was somewhat arbitrary, as higher BMI values in that study were also associated with abnormalities in the other somatic parameters. In line with the associations between MetS, depression (severity) and the role of inflammation, the overweight subgroup had the highest levels of psychopathology (Beijers et al., 2018). In contrast to our findings, however, the course of psychopathology did not differ between these groups (Beijers et al., 2018).

While our identified subgroup with both metabolic and inflammatory dysregulation scored significantly worse on all metabolic and inflammatory parameters compared to the healthy subgroup (except for LDL- and total cholesterol), the average level of inflammatory markers did not exceed the cut-off for low-grade inflammation applied in cardiovascular research (e.g. a hsCRP ⩾3; Pearson et al., 2003).

Additionally, the metabolic and inflammatory dysregulation subgroup more often suffered from dysthymia and had higher levels of anxiety at baseline, and consistently showed significantly higher depression severity levels over the follow-up period compared to the healthy subgroup. Thus, the small elevation in inflammation in this subgroup has great clinical consequences. Possibly, pathways involved in depression are highly sensitive to small changes in inflammation markers resulting in depression.

Although at younger age metabolic-inflammatory dysregulation seems to be related to atypical depression and the somatic-affective symptoms of depression, we did not find these associations in later-life. First, we did not find an association between depression subtypes (atypical/ melancholic) and a specific class. In adult depressed patients an association between atypical depression and metabolic-inflammatory dysregulation was found (Lamers et al., 2013; Milaneschi et al., 2017; Vogelzangs et al., 2014a, 2014b), nevertheless previous findings in older depressed patients were not consistent (Veltman et al., 2018; Vogelzangs et al., 2014a, 2014b). Therefore, distinction between atypical and melancholic depression in later life does not seem to be driven by metabolic-inflammatory dysregulation. Furthermore, in contrast to previous studies, higher depression severity levels were not associated with somatic depression symptoms (Capuron et al., 2008; Dantzer, O'Connor, Freund, Johnson, & Kelley, 2008; Duivis, Vogelzangs, Kupper, de Jonge, & Penninx, 2013; Marijnissen et al., 2013).

### Inflammatory depression

The severe inflammation subgroup was characterized by higher levels of hsCRP and IL-6 and the mild inflammation subgroup was characterized by elevated levels of NGAL and GDF-15. Patients in both subgroups were more obese and more often suffered from MetS compared to the healthy subgroup. Nonetheless, the level of inflammatory dysregulation in both subgroups was higher than can be expected based on solely the metabolic dysregulation. Therefore, the specific elevated levels of hsCRP and IL-6 in the severe inflammation subgroup may be due to an underlying primary inflammatory disease. Likewise, the elevated levels of NGAL and GDF-15 in the mild inflammation subgroup may represent the aging process, which can be accompanied by inflammatory dysregulation also known as ‘inflammaging’ (Franceschi et al., 2018), as this subgroup was the oldest, most physically inactive subgroup with the highest age of onset.

### Depression prognosis according to metabolic and inflammatory status

The healthy subgroup had a higher rate of depression remission at 2-year follow-up compared to the metabolic and inflammatory dysregulation subgroup. Furthermore, only the healthy subgroup continued to show a decrease in depression symptoms after the first half-year follow-up, whereas the other subgroups showed a deterioration of depression symptoms in the second half-year of follow-up. An explanation might be that patients were included after they had enlisted themselves for the start of depression treatment. Therefore, depression treatment might lose its effectiveness after the first half-year in patients with metabolic and/or inflammatory dysregulation. Chronic inflammation might thus represent a continuous vulnerability that averts long-term stabilization. A meta-analysis found that treatment targeting the inflammatory dysregulation in depression as an add-on to antidepressant treatment has beneficial effects on the depression of patients with MDD (Köhler-Forsberg et al., 2019). Examples of this treatment are non-steroidal anti-inflammatory drugs (NSAIDs) and statins. Nonetheless further research is necessary to understand the mechanism responsible for this anti-depressant effect. It is unclear whether the positive effects of anti-inflammatory treatment are caused by direct involvement in the depression cascade, or that the effect may be mediated by effects on somatic disease. Furthermore, effects were small, which suggests that only specific subgroups might benefit from augmentation with anti-inflammatory drugs. The subgroups with inflammatory dysregulation identified by our study might therefore be of particular interest for further research on anti-inflammatory therapy in the treatment of depression. These findings are in correspondence with previous studies in which both metabolic dysregulation and inflammation were found to be associated with worse depression outcomes in younger patients and cardiac patients (Capuron et al., 2008; Duivis et al., 2013; Howren et al., 2009; Marazziti, Rutigliano, Baroni, Landi, & Dell'Osso, 2014).

### Strengths and limitations

Strengths of this prospective study are the substantial sample size with availability of several clinical, and immune and metabolic parameters for LLD patients. Nonetheless, the severe and mild inflammation subgroups were relatively small subgroups with higher missings at 2-year follow-up. This could have led to attrition bias and limited statistical power to identify small but possibly meaningful differences between the classes. Moreover, the two extremely small classes, based on an extreme value of only one specific parameter, could not be further explored reliably. These subgroups might represent relatively rare cases with a specific underlying condition associated with a specific inflammatory profile. Finally, the subgroups may differ in the treatment received during the follow-up period. The data collected in the NESDO study did not allow us to control the analyses for these potential differences. However, at baseline the four subgroups did not differ in the medication treatment received.

## Conclusion

Nearly half of the patients with LLD showed metabolic-inflammatory dysregulation that was associated with more severe depression and worse depression prognosis. Future studies should examine whether the largest group with an overall, but relatively mild level of dysregulation would benefit from augmentation with anti-inflammatory drugs like NSAIDs or statins.

## Impact statement

We certify that this work is novel clinical research. The potential impact of this research on clinical care and health policy includes the following: as nearly half of the patients with late-life depressions suffer from metabolic-inflammatory dysregulation which is also associated with more severe depression and worse prognosis, a metabolic-inflammatory targeted treatment may benefit depressed older patients.
